# Structural brain changes in patients with persistent headache after COVID-19 resolution

**DOI:** 10.1007/s00415-022-11398-z

**Published:** 2022-09-30

**Authors:** Álvaro Planchuelo-Gómez, David García-Azorín, Ángel L. Guerrero, Margarita Rodríguez, Santiago Aja-Fernández, Rodrigo de Luis-García

**Affiliations:** 1 Laboratorio de Procesado de Imagen (LPI), Universidad de Valladolid, 47011 Valladolid, Spain; 2 Cardiff University Brain Research Imaging Centre (CUBRIC), Cardiff University, Cardiff, CF24 4HQ UK; 3 Department of Neurology, Headache Unit, Hospital Clínico Universitario de Valladolid, Avenida Ramón y Cajal, 3, 47003 Valladolid, Spain; 4 Department of Medicine, Universidad de Valladolid, 47005 Valladolid, Spain; 5 Department of Radiology, Hospital Clínico Universitario de Valladolid, 47003 Valladolid, Spain

**Keywords:** COVID-19, Headache, Gray matter, Diffusion tensor imaging, Migraine

## Abstract

Headache is among the most frequently reported symptoms after resolution of COVID-19. We assessed structural brain changes using T1- and diffusion-weighted MRI processed data from 167 subjects: 40 patients who recovered from COVID-19 but suffered from persistent headache without prior history of headache (COV), 41 healthy controls, 43 patients with episodic migraine and 43 patients with chronic migraine. To evaluate gray matter and white matter changes, morphometry parameters and diffusion tensor imaging-based measures were employed, respectively. COV patients showed significant lower cortical gray matter volume and cortical thickness than healthy subjects (*p* < 0.05, false discovery rate corrected) in the inferior frontal and the fusiform cortex. Lower fractional anisotropy and higher radial diffusivity (*p* < 0.05, family-wise error corrected) were observed in COV patients compared to controls, mainly in the corpus callosum and left hemisphere. COV patients showed higher cortical volume and thickness than migraine patients in the cingulate and frontal gyri, paracentral lobule and superior temporal sulcus, lower volume in subcortical regions and lower curvature in the precuneus and cuneus. Lower diffusion metric values in COV patients compared to migraine were identified prominently in the right hemisphere. COV patients present diverse changes in the white matter and gray matter structure. White matter changes seem to be associated with impairment of fiber bundles. Besides, the gray matter changes and other white matter modifications such as axonal integrity loss seemed subtle and less pronounced than those detected in migraine, showing that persistent headache after COVID-19 resolution could be an intermediate state between normality and migraine.

**Supplementary Information:**

The online version contains supplementary material available at 10.1007/s00415-022-11398-z.

## Background

Coronavirus disease 2019 (COVID-19) presents with a combination of respiratory and systemic symptoms, with headache being one of the most prominent features. Headache is described by approximately a quarter of patients [1, 2], with a phenotype that combines features of tension-type headache and migraine [3]. The presence of headache has been associated with a more efficient immune response [4, 5] and a better short-and-mid-term prognosis [6–8]. The median duration of headache is around 2 weeks [2], but in a fifth of patients, headache becomes persistent and adopts a chronic pattern, particularly if it persists 2 months after the acute phase [9].

In order to investigate on the biological underpinnings of persistent headache after COVID-19 resolution, magnetic resonance imaging (MRI) reveals itself as an ideal technique to assess the structural changes in the brain due to its associated good contrast of tissue and non-invasiveness. MRI data processing allows to analyze quantitative properties of the gray matter (GM) and white matter (WM). To assess GM changes, morphometry analysis from T1-weighted images allows the precise quantification of features such as the volume, cortical curvature, thickness and surface area of different cortical and subcortical GM areas [10]. In headache disorders, volume has been widely assessed [11], while area, curvature and thickness have been barely employed [12, 13]. On the other hand, diffusion MRI acquisitions allow the quantitative description of the WM properties. Diffusion tensor imaging (DTI) is commonly employed to model the diffusion MRI data, and white matter descriptors can be estimated from it. Fractional anisotropy (FA), mean diffusivity (MD), axial diffusivity (AD), and radial diffusivity (RD) are common choices. These metrics have been extensively used to study the presence of brain changes in patients with chronic headache disorders, especially migraine [14–18].

A few studies have assessed brain changes after COVID-19 resolution using MRI, none of them focused on persistent headache. In a study carried out 3 months after COVID-19 recovery, patients presented higher global GM volume, and, with regard to the WM, higher FA and lower MD, RD, and AD in comparison with healthy controls [19]. These trends reflect contrary patterns to what usually has been associated with atrophy or deterioration. In contrast, a large study with the UK Biobank composed of hundreds of longitudinal acquisitions of healthy controls and patients with previous COVID-19 infection has reported lower cortical thickness in the patient groups [20]. In another study with a 1-year follow-up, recovered patients presented a lower volume fraction of intracellular water than healthy controls, and decreased FA was found in patients who were admitted to an intensive care unit compared to those who were not admitted [21]. Finally, in a study assessing patients who recovered from COVID-19 accompanied by anosmia or hyposmia, the authors found lower FA and higher RD in recovered patients [22].

To date, there are no studies assessing structural changes in patients with persistent headache after COVID-19 resolution (hereinafter referred to as COV group). The main objective of this study is the evaluation and characterization of long-term brain structural changes in COV patients. In addition, a secondary objective is the comparison of these changes with those discovered in migraine, the headache disorder that has been most extensively studied using MRI data.

## Materials and methods

### Participants

An observational study with a case–control design, nested in a prospective cohort [2, 9], was conducted to assess the potential structural brain changes of COV patients. One the one hand, a group composed of healthy controls was used as a reference for a case–control study design. On the other hand, to better characterize the changes associated with headache, the COV patients were compared with a group composed of episodic migraine (EM), and another group composed of chronic migraine (CM) patients.

For the COV patients, the inclusion criteria were adapted from acute-phase studies [2]: (1) microbiologically confirmed COVID-19 diagnosis based on a real-time reverse-transcriptase-polymerase-chain-reaction (RT-PCR) assay from respiratory tract samples, or alternatively by the presence of anti-SARS-CoV-2 IgM + IgA antibodies, following the World Health Organization protocols [23, 24]; (2) new-onset headache presented during the acute phase of COVID-19, fulfilling criteria for acute headache attributed to systemic viral infection [25], that was not better accounted for another secondary headache disorder according to the International Classification of Headache Disorders, 3rd edition (ICHD-3) [25]; (3) age greater or equal to 18 years old; and also the following specific inclusion criteria: (4) persistence of headache for at least 3 months after the acute phase of COVID-19; (5) not resolution of the headache at the moment of MRI acquisition; (6) agreement to participate; (7) lack of use of drugs with potential effect on the central nervous system (better specified in exclusion criteria).

The study was conducted in the Headache Unit of Hospital Clínico Universitario de Valladolid (Valladolid, Spain), a third-level university public hospital. All consecutive patients that were infected from COVID-19 in Valladolid East health area between March 8, 2020, and April 11, 2020, were screened (*n* = 580 hospitalized patients and *n* = 1614 cases managed in an outpatient setting, out a population at risk of 162,431 inhabitants) [2] and prospectively followed up for at least 9 months [9]. All these patients and those that were referred to the headache outpatient clinic were assessed for eligibility. For the COV patients included in the sample, diverse clinical features of headache and the presence of anosmia were collected.

All the participants were aged between 18 and 60 years. Regarding the specific inclusion and exclusion criteria of the other three groups in this study (healthy controls, EM and CM), they are available elsewhere [18]. Briefly, the diagnosis of patients with EM and CM was based on the ICHD-3 criteria [25], and these patients presented a stable clinical situation with at least 1 year suffering from migraine. The exclusion criteria were common in all four groups and were: (1) death during follow-up; (2) unavailability to participate due to unstable medical condition; (3) prior history of cognitive impairment or dementia; (4) speech or language disorders that made headache evaluation impossible; (5) consent withdrawal to participate in the study; (6) use of therapeutic or illicit drugs with potential effects on the nervous system, including antidepressants, barbiturates, neuroleptics, antiepileptics, and opiates; (7) prior history of other primary or secondary headache disorders, excluding infrequent tension-type headache (less than one attack per month); (8) previous history of moderate-to-severe cranio-cervical trauma; (9) prior history of neurological or neurosurgical disorders other than COVID-19 (in the COV group); (10) suffering from other painful syndromes, or neurological or psychiatric disorders; (11) pregnancy or childbearing; (12) conditions that contraindicated an MRI acquisition; (13) claustrophobia; (14) presence of unexpected vascular malformation in MRI that could affect the morphological evaluation; (15) any clinical condition that avoided an accurate description of the headache phenotype. Both migraine patients and COV patients were excluded if they had used any prior preventive treatment before MRI acquisition. The patients with migraine were recruited after their first visit to the aforementioned Headache Unit and, in case of prescribed preventive treatment, it began just after the MRI acquisition in a period shorter than 1 month. Data and imaging from patients with migraine and controls were obtained before the beginning of the COVID-19 pandemic. The Ethics Review Board of Valladolid East health area approved this study (PI-GR-20-2017).

### MRI acquisition

T1- and diffusion-weighted images (DWI) were collected for the four groups of subjects. For the identification of possible abnormalities, T2-weighted and FLAIR images were additionally acquired from the COV group. All patients were scanned in the same MRI scanner, a Philips Achieva 3 T MRI unit (Philips Healthcare, Best, The Netherlands) with a 32-channel head coil.

T1-weighted images were acquired using a Turbo Field Echo sequence with the following parameters: repetition time (TR) = 8.1 ms, echo time (TE) = 3.7 ms, flip angle = 8º, 256 × 256 matrix size, spatial resolution of 1 × 1 × 1 mm^3^ and 160 sagittal slices covering the whole brain.

For the diffusion-weighted data, TR = 9000 ms, TE = 86 ms, flip angle = 90º, 61 diffusion gradient orientations, one baseline volume, *b*-value = 1000 s/mm^2^, 128 × 128 matrix size, spatial resolution of 2 × 2 × 2 mm^3^ and 66 axial slices covering the whole brain were the employed parameters.

The parameters for the acquisition of the T2-weighted images were: Turbo Spin Echo sequence, TR = 3000 ms, TE = 80 ms, flip angle = 90º, 560 × 560 matrix size, spatial resolution of 0.43 × 0.43 × 5 mm^3^, and 28 axial slices.

Finally, 3D high-resolution FLAIR images were obtained using TR = 4800 ms, TE = 308 ms, inversion time (TI) = 1650 ms, flip angle = 90º, 240 × 240 matrix size, spatial resolution of 0.56 × 1.04 × 1.04 mm^3^, and 321 sagittal slices to cover the whole brain.

### Image processing

The analysis was focused on the assessment of the GM and WM structure. In the following sections, the corresponding processing steps followed for the separate analysis of each type of tissue are described.

### Gray matter morphometry

The automatic cortical parcellation pipeline from the FreeSurfer software (v6.0.0) was applied to the T1-weighted images. The steps from this procedure included the segmentation of cortical and subcortical GM structures and the calculation of geometrical descriptors for further analysis. Specifically, the previous processing steps of the pipeline were skull stripping, Talairach transformation, segmentation of subcortical gray and white matter, including the boundary tessellation of both tissue types, intensity normalization, and surface deformation [26–29]. The pipeline was used to extract the mean cortical curvature, thickness, surface area and GM volume of 68 (34 bilateral) cortical regions. In addition, GM volume was computed for seven bilateral regions and the bilateral cerebellum (16 regions). This yields a total of 288 morphometric features for the GM.

### Diffusion MRI processing

First, DWIs were preprocessed. The preprocessing steps were denoising following the Marchenko-Pastur Principal Component Analysis procedure, Gibbs ringing removal, correction for eddy currents, motion and B1 field inhomogeneities. These steps were carried out with the “dwidenoise”, “mrdegibbs”, “dwifslpreproc” and “dwibiascorrect” tools from MRtrix (version 3.0.2) [30–36]. Once the preprocessing was finished, a whole brain mask excluding the skull was extracted using the “dwi2mask” tool from MRtrix [37].

After preprocessing, diffusion tensor fitting was performed on the DWIs using least squares, and fractional anisotropy (FA), mean diffusivity (MD), and axial diffusivity (AD), were obtained as WM descriptors using the “dtifit” tool from FSL software suite (version 5.0.9) [38]. An additional descriptor, the radial diffusivity (RD) was also calculated as the average of the second and third eigenvalues from the diffusion tensor, also obtained with “dtifit”.

For the posterior statistical analysis, the aforementioned diffusion parameters were compared in diverse WM tracts. Specifically, tract-based spatial statistics (TBSS) was used as a procedure to define a WM skeleton where the values from the diffusion parameters are assessed [39]. Briefly, the TBSS method consists of a non-linear registration of the FA images to a template in the Montreal Neurological Institute (MNI) space with the FNIRT and FLIRT tools from FSL. The registered images were averaged, and a mean FA skeleton of the WM tracts was generated using a FA value of 0.2 as threshold to distinguish WM from GM. The TBSS process was repeated for the non-FA parameters using the FA registration as reference. To identify specific WM regions, the JHU ICBM-DTI-81 White-Matter Atlas was used [40, 41]. Moreover, the minimum volume per region to consider statistically significant results was 30 mm^3^.

### Statistical analysis

The main objective of the analysis was the comparison of the parameters of the COV patients with healthy controls (HC). To better characterize the properties of the COV group, these patients were compared against EM, and CM. The detailed comparisons of GM and WM diffusion descriptors between the two migraine groups and HC is available elsewhere [12, 18, 42].

Two-by-two group comparisons of age and sex were carried out using the Mann–Whitney *U* test and Fisher’s exact test, respectively.

With regard to the comparison of the GM morphometry parameters between the COV and HC groups, data were tested for normal distribution with the Shapiro–Wilk test. If this assumption was not met, a Mann–Whitney *U* test was applied. Furthermore, the homogeneity of variance assumption was tested by the Levene test. If normality assumption was met, a t-test was used to compare the values between two groups, considering equal or different variance depending on the results of the Levene test. An analysis of covariance (ANCOVA) was always used to compare GM volume values considering intracranial volume as covariate of no interest. For any secondary comparison with the EM or CM groups, an ANCOVA was employed including age as covariate of no interest. As secondary assessment, sex was also added as covariate of no interest to the ANCOVA. In addition, for the main comparisons between COV and HC, results were also corrected for age and sex following the same ANCOVA procedure conducted in the comparison between COV and the migraine groups. The Benjamini–Hochberg False Discovery Rate (FDR) procedure was applied to correct the results for multiple comparisons [43]. The threshold for statistical significance was set at *p* < 0.05.

Regarding the TBSS analysis of the diffusion descriptors, the “randomise” tool from FSL with the threshold-free cluster enhancement (TFCE) option was employed to determine the differences between the groups of interest [44, 45]. Briefly, this tool is used to perform a permutation test. We employed 5000 permutations and established the statistical threshold for statistical significance at *p* < 0.05 after applying a family-wise error (FWE) correction for multiple comparisons. For the comparisons between the COV group and the EM and CM groups, considering that patients from the first group are, on average, older than patients with migraine, results were age-corrected. As a secondary analysis, in the comparison between COV and the other three groups, results were additionally corrected for sex.

In addition, to assess the magnitude of the identified differences of the morphometry and diffusion parameters, the Cohen’s d was computed for the comparisons with statistically significant differences. The values employed to assess each parameter and specific region were those included in the Desikan–Killiany atlas for morphometry, and the skeleton and area of each region according to the JHU ICBM-DTI-81 atlas for diffusion parameters. The pooled standard deviation was computed as the squared root of the addition of the variance of each assessed group multiplied by the pertinent sample size minus one divided by the total sample size of the two groups minus two. The final Cohen’s *d* value was the difference between the mean value of the two groups divided by the pooled standard deviation.

To assess the relationship between clinical parameters of COV patients and gray and white matter descriptors, Spearman’s rank correlation coefficient was employed. The clinical variables were the disease duration and the headache frequency. The values of the gray matter morphometry parameters were the same as those used for the statistical comparisons between groups. For the white matter descriptors, a ROI-based approach was carried out. Individual label maps for each subject were extracted applying the inverse warp fields of the registration of the FA images described for the TBSS procedure to the JHU ICBM-DTI-81 atlas. For the gray matter and white matter results, only the regions and parameters with statistically significant differences between COV patients and any other group were considered.

## Results

**Fig. 1:**
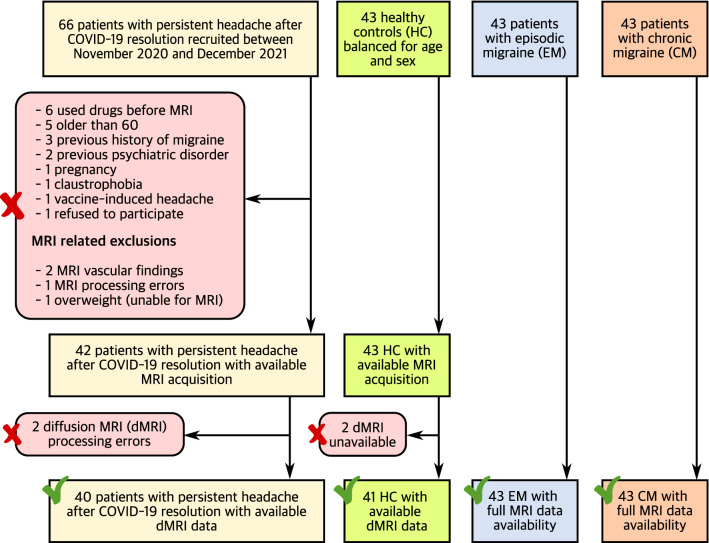
Flowchart of the study. The number of patients with persistent headache after COVID-19 resolution and healthy controls is different in the gray matter and white matter assessments due to registration errors with the diffusion images or unavailable diffusion MRI acquisitions

In total, 66 COV patients were initially recruited. The final sample was composed of 42 COV patients and 43 HC balanced for age and sex. Median time between onset of headache attributed to COVID-19 and MRI acquisition in COV patients was 10 months (range 3–20 months). In addition, 43 patients with EM, and 43 patients with CM were considered. Two patients for each of the first two groups were discarded for the diffusion MRI analysis due to registration errors. The study flowchart detailing the sample at each stage is shown in Fig. 1. The two-by-two comparisons of the demographic characteristics between the COV group and the other three groups are available in Table 1. Further information about specific clinical characteristics related to headache in COV patients, i.e., phenotype, location, mean intensity, quality, and symptoms during headache, together with the presence of anosmia, is available in Table 2.

**Table 1:** Clinical and demographic characteristics of patients with persistent headache after COVID-19 resolution, healthy controls (HC), episodic migraine (EM) and chronic migraine (CM) patients

	COVID-19 headache (*n* = 42)	HC (*n* = 43)	EM (*n* = 43)	CM (*n* = 43)	Statistical test
Gender, male/female	11/31 (26/74%)	11/32 (26/74%)	9/34 (21/79%)	6/37 (14/86%)	1. *p* = 1^**†**^ 2. *p* = 0.62^**†**^ 3. *p* = 0.18^**†**^
Age (years)	43.8 ± 10.2	41.8 ± 10.2	40.1 ± 6.4	41.3 ± 7.1	1. *U* = 1015, *p* = 0.33^‡^ 2. *t* = 2.00, *p* = 0.049^§^ 3. *U* = 1110, *p* = 0.070^‡^
Disease duration	10.1 ± 3.4 months		16.0 ± 11.4 year	21.9 ± 10.2 year	
Headache frequency (days/month)	30 ± 0		3.5 ± 1.9	22.8 ± 6.7	
Diffusion MRI sample	*n* = 40	*n* = 41	*n* = 43	*n* = 43	
Gender, male/female	11/29 (28/72%)	11/30 (27/73%)	9/34 (21/79%)	6/37 (14/86%)	1. *p* = 1^**†**^ 2. *p* = 0.61^**†**^ 3. *p* = 0.17^**†**^
Age (years)	43.7 ± 10.3	41.0 ± 9.6	40.1 ± 6.4	41.3 ± 7.1	1. *U* = 959, *p* = 0.19^‡^ 2. *t* = 1.90, *p* = 0.062^§^ 3. *U* = 1050, *p* = 0.084^‡^
Disease duration	10.2 ± 3.5 months		16.0 ± 11.4 year	21.9 ± 10.2 year	
Headache frequency (days/month)	30 ± 0		3.5 ± 1.9	22.8 ± 6.7	

^**†**^Fisher’s exact test. ^‡^Mann–Whitney *U* test. ^§^Two-tailed, unpaired Student’s *t*-test (Welch test). 1. COVID-19 headache vs. HC. 2. COVID-19 headache vs. EM. 3. COVID-19 headache vs. CM. Data are expressed as means ± SD. The phenotype other does not adhere to migraine or TTH following ICHD-3 guidelines. TTH = tension-type-headache

**Table 2:** Specific clinical characteristics related to headache of patients with persistent headache after COVID-19 resolution

Headache characteristic	Full sample (*n* = 42)	Diffusion MRI sample (*n* = 40)
Phenotype, migraine/TTH/other	20/13/9 (47.6/31.0/21.4%)	20/12/8 (50/30/20%)
Mean intensity (1–10)	7.3 ± 1.6	7.4 ± 1.6
Anosmia	22 (52.4%)	21 (52.5%)
Location		
Hemicranial	10 (23.8%)	9 (22.5%)
Holocranial	31 (73.8%)	30 (75%)
Quality		
Pressing	30 (71.4%)	28 (70%)
Throbbing	20 (47.6%)	20 (50%)
Burning	1 (2.4%)	1 (2.5%)
Symptoms during headache		
Photo- and phonophobia	21 (50%)	20 (50%)
Nausea or vomiting	16 (38.1%)	16 (40%)
Aggravated by movement	22 (52.4%)	21 (52.5%)

Mean intensity was expressed as mean ± SD. The phenotype other does not adhere to migraine or TTH following ICHD-3 guidelines. TTH = tension-type-headache. Anosmia was not a characteristic directly related to headache, but it was included due to its relationship with COVID-19

### Gray matter morphometry parameters

#### Comparison between COV and HC

**Fig. 2:**
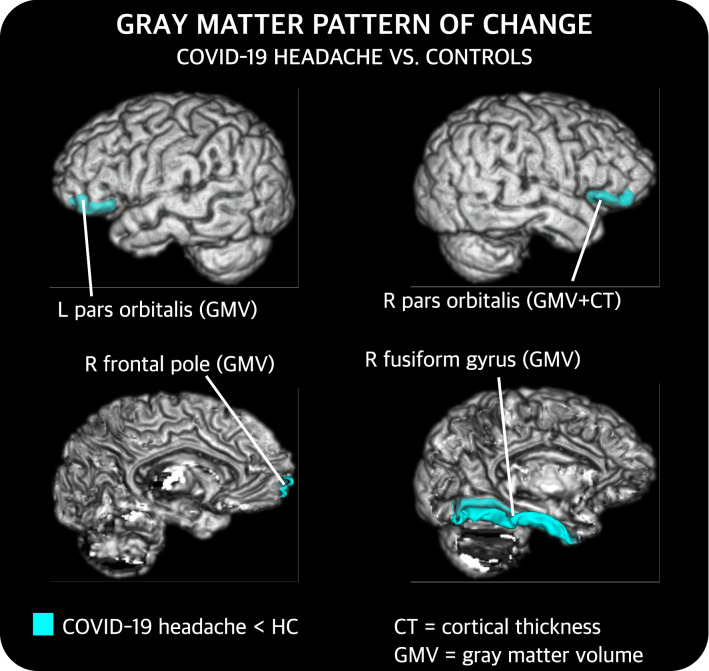
Gray matter regions that present statistically significant changes between patients with persistent headache after COVID-19 resolution (COV) and healthy controls (HC). After false discovery rate correction, COV patients showed lower gray matter volume (GMV) and cortical thickness (CT) than HC. *L* left, *R* right. These results were also statistically significant after correcting for age and sex. Additional results after this correction are not shown

Compared to HC, COV patients showed statistically significant lower GM volume in the bilateral pars orbitalis (corrected *p* = 0.029 left; corrected *p* = 0.033 right), and the right fusiform gyrus (corrected *p* = 0.033) and frontal pole (corrected *p* = 0.033). Moreover, patients also presented lower cortical thickness than HC in the right pars orbitalis (corrected *p* = 0.008). No statistically significant differences were found in cortical curvature or surface area. The location of these findings is graphically shown in Fig. 2. After the correction for age and sex, these results were statistically significant, and, additionally, lower cortical thickness values were found in COV compared to HC in the left rostral anterior cingulate gyrus (corrected *p* = 0.024).

#### Comparison between COV and migraine

Regarding the differences between COV and EM patients after correcting for age, significantly higher cortical thickness (corrected *p* = 0.012) in the left paracentral cortex, and lower subcortical volume values in the left accumbens (corrected *p* = 0.022) and the right thalamus (corrected *p* = 0.025) were observed in the COV group. No statistically significant differences were identified in cortical curvature or surface area. The regions with statistically significant differences were the same after additionally correcting the results for sex.

With respect to the comparison between COV and CM patients after correcting for age, statistically significant differences were found for every parameter except the surface area. First, lower cortical curvature values in the left cuneus (corrected *p* = 0.007) and the right precuneus (corrected *p* = 0.028) were observed in COV patients. Also, higher cortical GM volume in the left caudal middle frontal gyrus (corrected *p* = 0.012), paracentral cortex (corrected *p* = 0.032) and posterior cingulate gyrus (corrected *p* = 0.038) were found in COV patients. Moreover, higher cortical thickness in the left banks of the superior temporal sulcus (corrected *p* = 0.007) and paracentral cortex (corrected *p* = 0.007) were identified in COV patients. The regions with statistically significant differences remained the same after additionally correcting the results for sex.

**Fig. 3:**
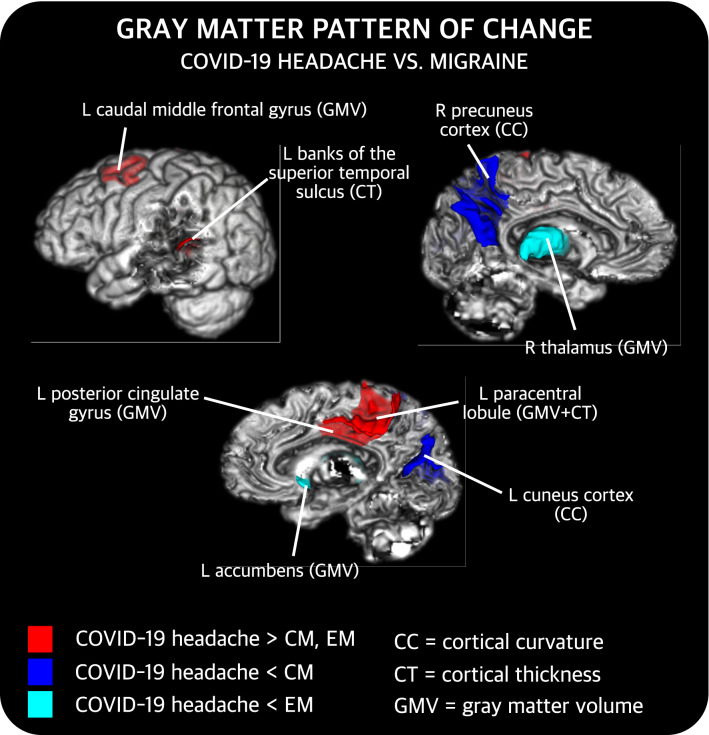
Gray matter regions that present statistically significant changes between patients with persistent headache after COVID-19 resolution (COV) and patients with episodic migraine (EM) and/or chronic migraine (CM). After false discovery rate correction, COV patients showed higher cortical gray matter volume (GMV) and cortical thickness (CT) than EM and CM, lower subcortical GMV than EM, and lower cortical curvature (CC) than CM. *L* left, *R* right

**Fig. 4:**
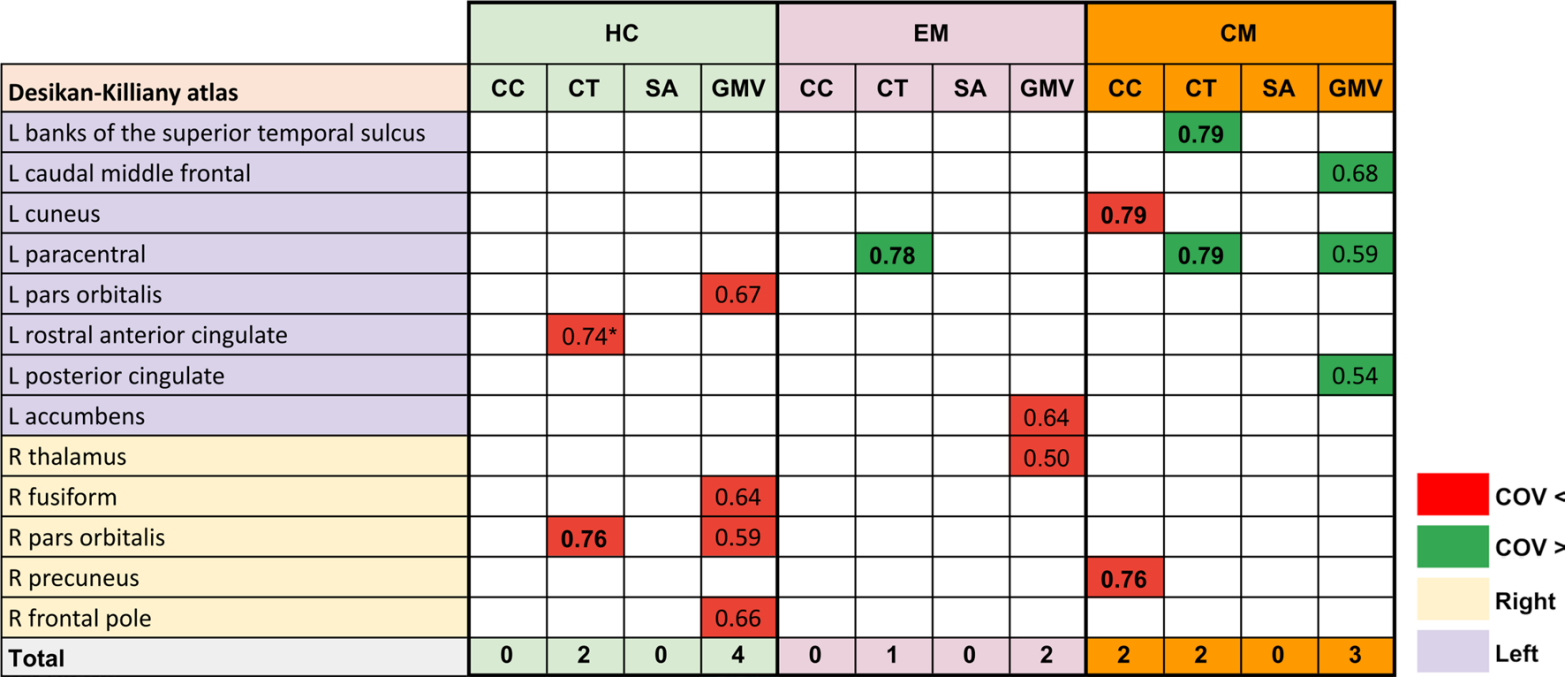
Cohen’s d values of the comparisons with statistically significant differences of gray matter morphometry parameters. Cells in red reflect comparisons with lower values in patients with persistent headache after COVID-19, while cells in green reflect the opposite trend. *Results obtained exclusively when adding sex as a covariate

A summary of these results can be found in Fig. 3, and detailed results considering all groups are available in the Supplementary material (Tables S1–3). Regarding the effect size of the comparisons with significant differences, all the values were in the range of medium effect size (0.5 ≤ *d* < 0.8), as shown in Fig. 4.

#### Comparison between patients with migraine and HC

The results of the comparisons between CM, EM and HC, which are not the focus of this study, have been published elsewhere, employing a higher sample size and balancing age and sex according to the migraine patients’ characteristics [12]. To better understand the results of this research, we here summarize these results. Briefly, cortical curvature was higher and cortical thickness, surface area and cortical GM volume were lower in both migraine groups compared to HC. Curvature values were higher in migraine patients in regions such as the cingulate gyrus, lateral occipital cortex, the precuneus and the paracentral cortex. Cortical thickness was lower in patients, with higher level of significance, in the inferior temporal and fusiform gyri. GM volume and surface area were commonly lower in patients in the insula, the superior temporal gyrus, pars triangularis and pars orbitalis, and additionally lower area values were found in the precuneus, cingulate gyrus, supramarginal gyrus and diverse frontal, temporal and parietal gyri. Moreover, GM volume and widespread surface area regions presented lower values in CM compared to EM, in contrast to cortical thickness, for which higher values were identified in CM in the inferior temporal gyrus.

### Diffusion parameters in white matter

#### Comparison between COV and HC

**Fig. 5:**
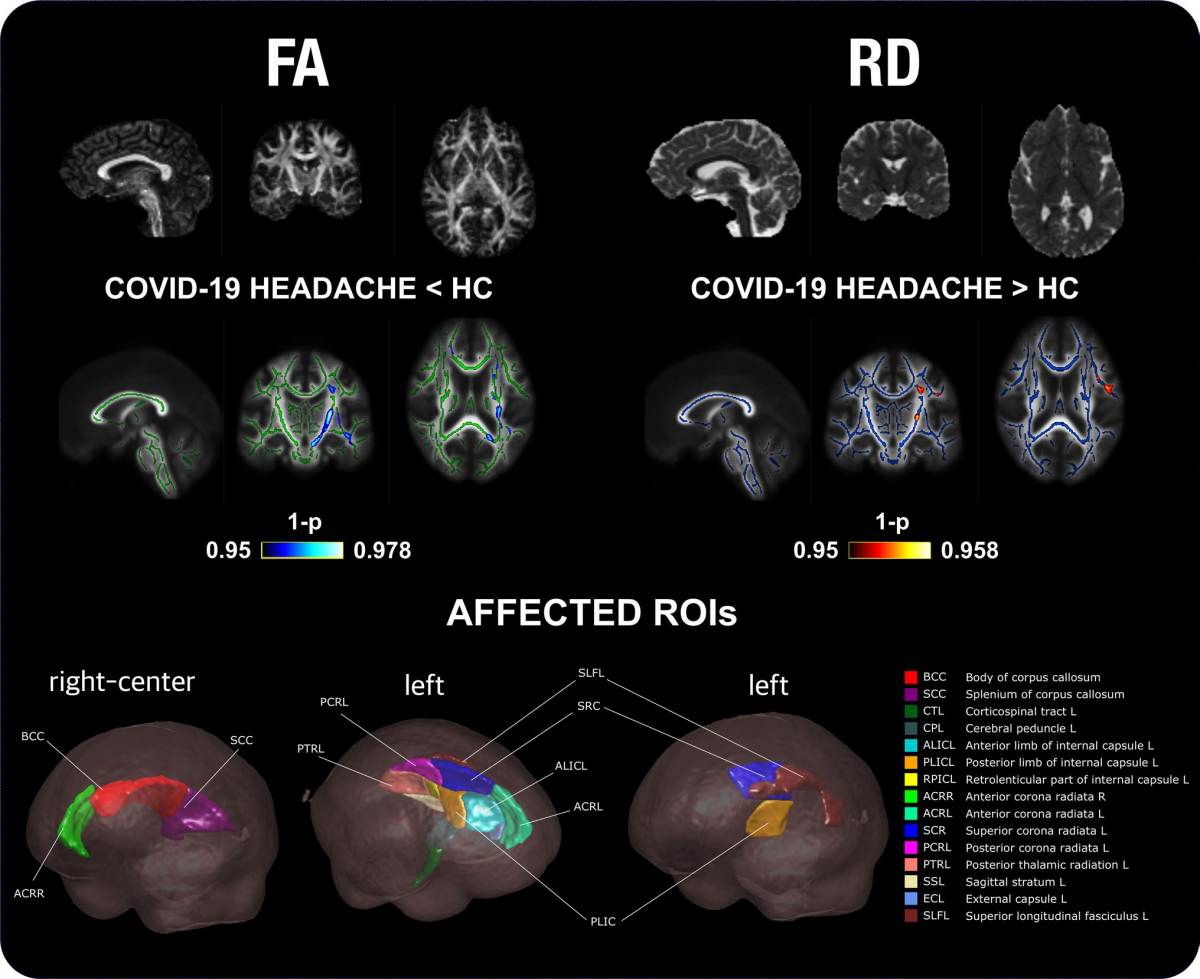
TBSS results of the diffusion descriptors that showed statistically significant differences in patients with persistent headache after COVID-19 resolution (COV) compared to healthy controls (HC). COV patients presented lower fractional anisotropy (FA) and higher radial diffusivity (RD) values than HC. White matter skeleton is shown in blue for the RD comparison, and in green for the FA comparison. Voxels with statistically significant differences are shown in red-yellow for the RD comparison (higher values in COV), and in blue for the FA comparison (lower values in COV) considering the assessment without adding sex as a covariate. The color bar shows the 1-*p* values (family-wise error corrected). At the bottom, the regions of interest (ROIs) with FA or RD significant differences are shown

Statistically significant lower FA was found in COV compared to HC in 15 regions, and in 7 additional regions after correcting for sex. These regions were the internal and external capsule, the cerebral peduncle, the corticospinal tract, the bilateral corona radiata, the corpus callosum, the thalamic radiation, the superior longitudinal fasciculus, and the superior fronto-occipital fasciculus. In addition, higher RD values were found in COV patients in three regions from the left hemisphere, and in eight additional left regions after correcting for sex: superior longitudinal fasciculus, corona radiata, the external and internal capsule, the cerebral peduncle, and the sagittal stratum. These FA and RD differences are shown in detail in Fig. 5, showing the differences for each region in Tables S4–7.

**Fig. 6:**
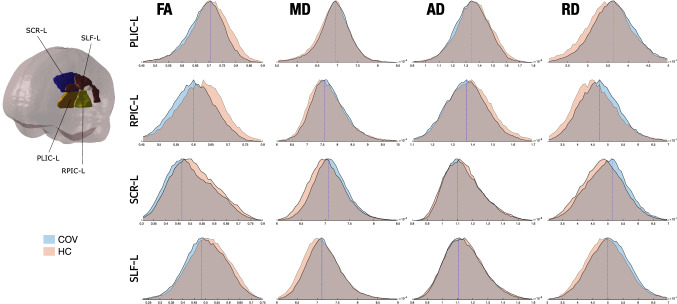
Distribution of the diffusion tensor parameters in patients with persistent headache after COVID-19 resolution (COV) and healthy controls (HC). The distributions of the three regions with RD statistically significant differences between COV and HC, and the one for the left retrolenticular part of the internal capsule (RPIC-L), a representative region, are shown. COV patients showed lower FA and higher RD values than HC, and also non-significant lower MD and higher AD. *PLIC-L* left posterior limb of internal capsule, *SCR-L* left superior corona radiata, *SLF-L* left superior longitudinal fasciculus

**Fig. 7:**
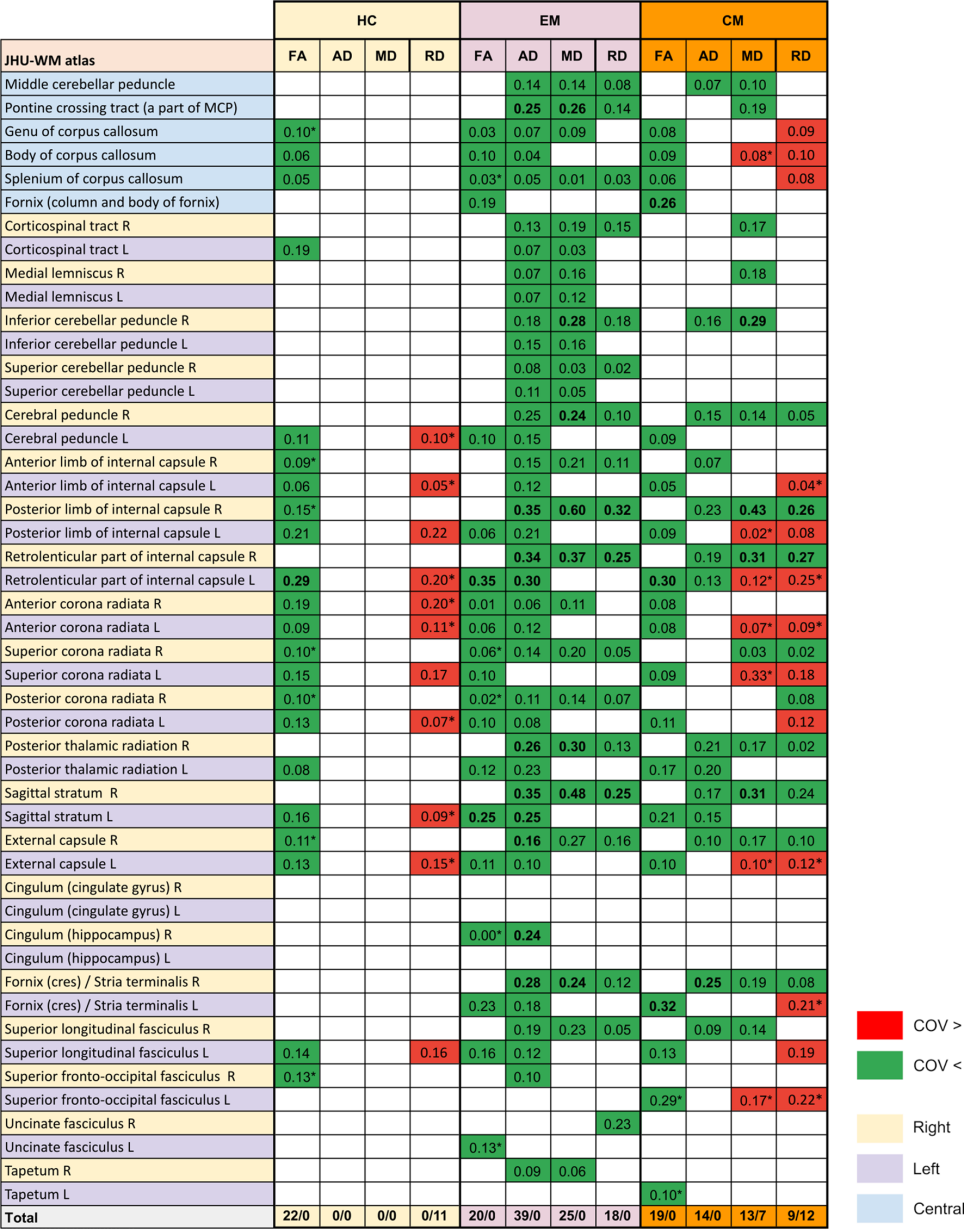
Cohen’s d values of the comparisons with statistically significant differences of diffusion parameters. Cells in green reflect comparisons with lower values in patients with persistent headache after COVID-19, while cells in red reflect the opposite trend. The 48 regions from the JHU ICBM-DTI-81 atlas are shown. *Results obtained exclusively when adding sex as a covariate

Figure 6 shows a graphical depiction of these WM changes. The distribution of the values of the four DTI parameters for COV and HC groups is shown for the three regions with RD significant differences and the left retrolenticular part of internal capsule. These four regions are represented to observe the distribution of the values of all the RD differences and the region with the highest effect size in the FA comparison, to better appreciate the FA differences between groups. In order to gain insight about the direction and magnitude of these differences, Fig. 7 presents the presence or absence of statistically significant changes for each WM region, together with their direction and Cohen’s *d*. The effect size of the identified differences was very low (*d* < 0.2) except for comparisons involving the internal capsule.

#### Comparison between COV and migraine

Widespread statistically significant differences were found between the COV group and patients with EM and CM considering the four WM descriptors that were employed (Fig. 7).

Regarding FA, COV patients showed lower values than EM and CM in 15 (also in five additional regions after correction for sex) and 17 regions (also in one additional region after correction for sex), respectively. For all FA comparisons, the main regions with differences were the corpus callosum, bilateral corona radiata, and left external and internal capsule, left cerebral peduncle, and left thalamic radiation, finding additional differences in the left superior longitudinal fasciculus in the comparison with EM.

With respect to the AD, COV patients presented statistically significant lower values in comparison with EM in 39 regions (same number after correcting for sex), in 14 regions compared to CM, mostly in the right hemisphere, and in 8 out of these 14 regions after correcting for sex. The main bilateral regions where differences against CM and EM were observed were the cerebellar and cerebral peduncles, the internal capsule, and the thalamic radiation. Moreover, differences against EM were also found in the corpus callosum, corona radiata, corticospinal tract, superior longitudinal fasciculus, external capsule, and thalamic radiation.

Statistically significant lower MD values were found in COV patients with respect to EM, in 25 regions (in three regions less after correction for sex), and CM, in 14 regions (in five regions less after correction for sex). Most differences were found in the right hemisphere. Moreover, only after the correction for sex, higher MD values in COV patients compared to CM were detected in the body of corpus callosum and left hemisphere regions such as the internal and external capsule, the corona radiata, and the superior longitudinal fasciculus.

With regard to RD, lower values were found in COV patients with respect to EM in 18 regions (in three regions less after correcting for sex), mostly in the right hemisphere. In the comparison with CM patients, on the one hand, lower RD was found in nine regions. On the other hand, increased RD was detected in six different regions, and in six additional regions after correcting for sex. The regions with higher RD were the corpus callosum, and the left corona radiata and superior longitudinal fasciculus, the internal and external capsule, the corona radiata, the fornix, and the superior fronto-occipital fasciculus. The lower MD and RD values were identified particularly in the external and internal capsule, thalamic radiation, corticospinal tract, corona radiata, superior longitudinal fasciculus and cerebellar peduncle. Additional differences for EM were found in the corpus callosum, and for CM in the cerebral peduncle.

**Fig. 8:**
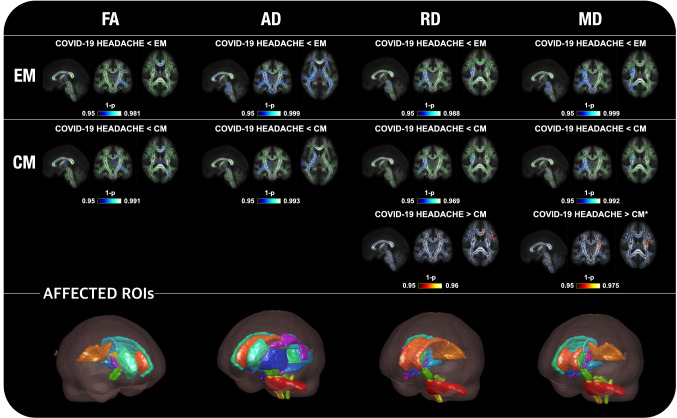
TBSS results of the diffusion descriptors that showed statistically significant differences in patients with persistent headache after COVID-19 resolution (COV) compared to patients with episodic migraine (EM) and chronic migraine (CM). COV patients presented lower fractional anisotropy (FA), axial diffusivity (AD), mean diffusivity (MD) and radial diffusivity (RD) values than EM and CM. Moreover, higher RD and MD values in COV patients compared to CM were also found. White matter skeleton is shown in blue for these last RD and MD comparisons, and in green for the remaining comparisons. Voxels with statistically significant differences are shown in red-yellow for the RD comparison with higher values in COV, and in blue for the comparisons with lower values in COV. The color bar shows the 1-*p* values (family-wise error corrected). At the bottom, the regions of interest (ROIs) with significant differences are shown for each parameter considering the assessment without adding sex as a covariate. *Results obtained exclusively when adding sex as a covariate

**Fig. 9:**
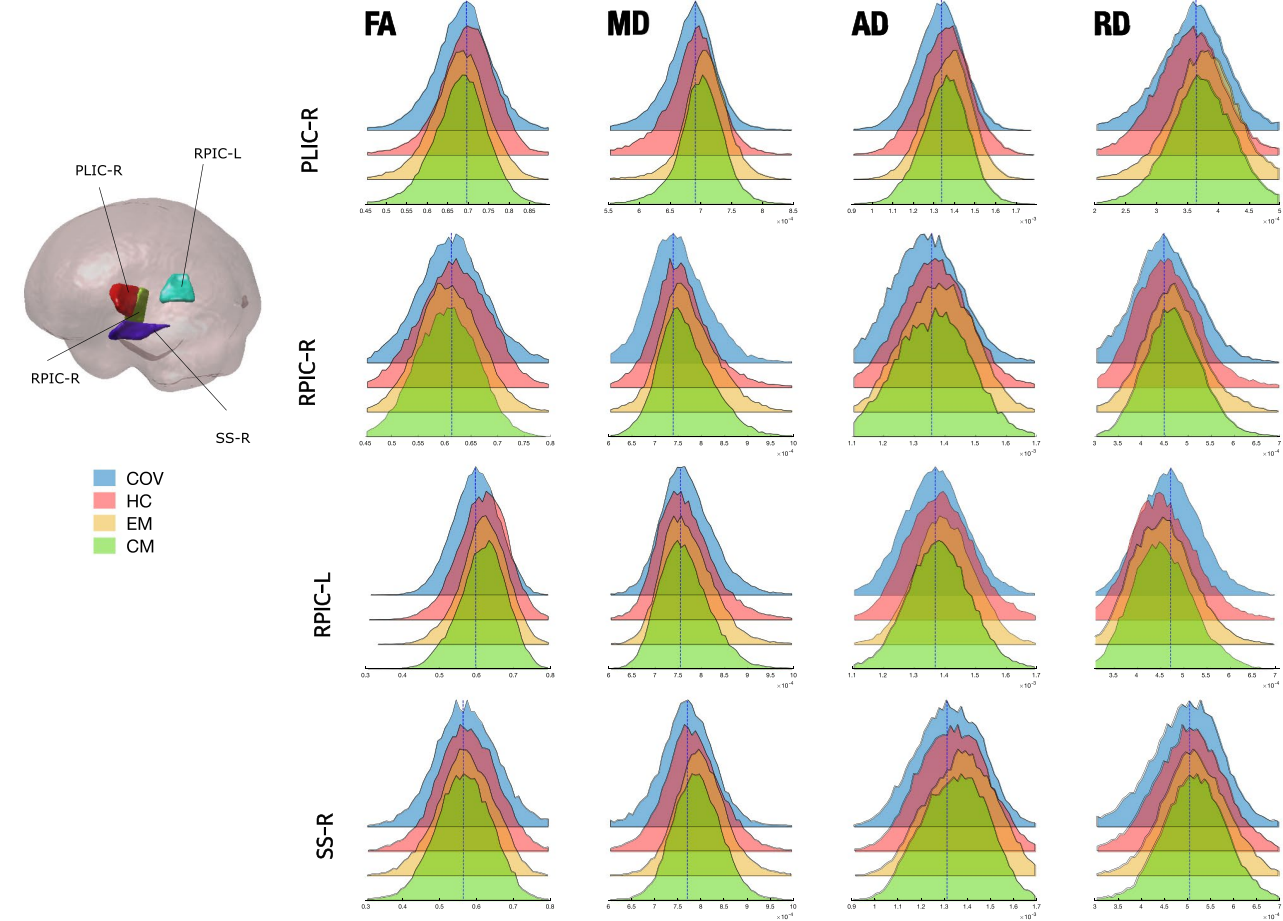
Distribution of the diffusion tensor parameters in patients with persistent headache after COVID-19 resolution (COV), healthy controls (HC) and patients with episodic migraine (EM) and chronic migraine (CM). The distributions of four representative regions of interest (ROIs), right and left retrolenticular part of internal capsule (RPIC-R/L; ROI3 and ROI2, respectively), right posterior limb of internal capsule (PLIC-R; ROI1), and right sagittal stratum (SS-R; ROI4), are shown. For internal capsule values, COV patients showed left-skewed FA and right-skewed RD distributions. MD, AD and RD distributions of right hemisphere regions were left-skewed in COV patients compared to CM and EM

These results are graphically depicted in Fig. 8, and details of the direction and effect sizes of the significant changes are provided in Fig. 7. The differences for each region are shown in Tables S8–20. In Fig. 9, a comparison of the distribution of the values between COV patients, HC, EM and CM is shown for the right posterior limb of internal capsule, bilateral retrolenticular part of internal capsule, and the right fornix–stria terminalis (region with AD, MD and RD differences compared to both migraine groups). These four regions are represented in Fig. 9 to better appreciate the distribution of the values of the differences between COV patients and both migraine groups, as these regions presented the highest effect size in the comparisons between these groups for the four diffusion descriptors (Fig. 7). Figure 9 shows that FA distributions were similar for the four groups considering the right hemisphere values. However, COV patients presented a left-skewed distribution compared to the other three groups, which presented similar distributions except for the right posterior limb of internal capsule, which was right-skewed for the HC. For the MD, AD and RD distributions of right hemisphere regions, the distributions for COV patients were left-skewed in comparison to the distributions for both migraine groups.

Regarding the effect size, it was very low (*d* < 0.2) for most comparisons between COV patients and both EM and CM groups. However, some regions showed low (0.2 < *d* < 0.5) or even medium effect size (0.5 < *d* < 0.8) in the comparison with EM, and sometimes also with CM. Regions with low or medium effects size for any of the four parameters were the internal capsule, inferior cerebellar peduncle, pontine crossing tract, fornix, thalamic radiation, cingulum, sagittal stratum, superior longitudinal fasciculus and uncinate fasciculus. All the Cohen’s d values are shown in Fig. 7.

#### Comparison between patients with migraine and HC

Comparisons between the two migraine groups and HC, which are not the focus of this paper, were performed in previous studies, using a sample size slightly higher than that in the present study [18, 46]. We next summarize those findings for the sake of readability. Briefly, considering the DTI parameters, AD and MD values were lower in CM compared to EM in 40 and 38 regions, respectively. The main regions with differences between both migraine groups were the cerebellar and cerebral peduncles, the superior longitudinal fasciculus, corpus callosum, corona radiata, external and internal capsules, thalamic radiation and corticospinal tract. No further differences were identified without considering covariates of no interest. Including additional covariates of no interest such as migraine duration or time from onset of CM, higher AD values were identified in EM compared to HC, and lower FA values in CM compared to HC. Higher AD values in EM were found in regions such as the superior corona radiata, external and internal capsule, posterior thalamic radiation, sagittal stratum and cerebral peduncle. Lower FA values in CM were reported in the superior cerebellar peduncle, corpus callosum, corona radiata, external and internal capsule, sagittal stratum and fornix.

### Correlation analysis

**Fig. 10:**
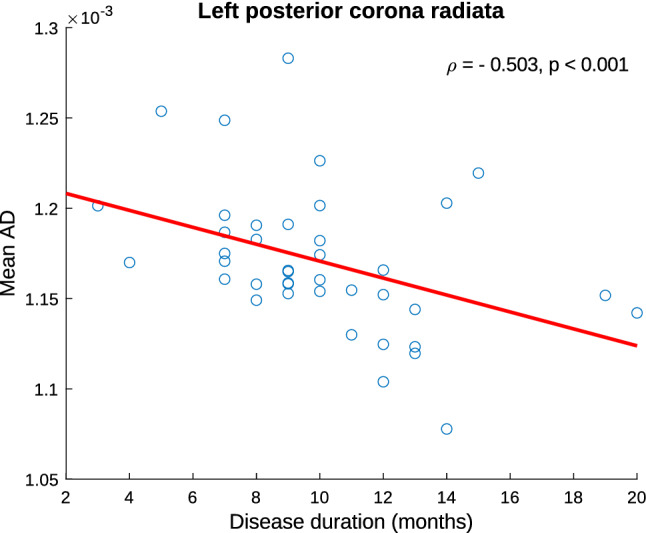
Association graph between clinical parameters and white matter diffusion descriptors in patients with persistent headache after COVID-19 resolution. A significant negative correlation was found between disease duration and mean AD

The assessment of the headache frequency in COV patients was not conducted because all the patients suffered from daily headache, i.e., they had the same frequency value. Regarding the correlation with gray matter morphometry parameters, no statistically significant results were found in COV patients. In contrast, a significant negative correlation was found between the mean AD values and the disease duration in COV patients (*ρ* = − 0.503, *p* < 0.001) in the left posterior corona radiata, as shown in Fig. 10. No significant correlations were identified for mean FA, RD or MD.

A correlation analysis of the migraine patients with a higher sample size can be found elsewhere [12, 18]. Briefly, in EM patients, significant negative correlations were found between disease duration and GM volume in the pars opercularis, superior frontal gyrus, and insula, and between disease duration and surface area in the insula. For both migraine groups, no significant correlations were found for headache frequency. No significant correlation between diffusion measurements in white matter and total migraine duration was detected. No statistically significant correlations were identified for CM patients.

## Discussion

In this study, we investigated the structural GM and WM changes in patients with persistent headache after COVID-19 resolution using T1-weighted and diffusion MRI data.

COV patients showed lower GM volume in the bilateral pars orbitalis, right fusiform gyrus and frontal pole, and lower cortical thickness in the right pars orbitalis when compared to HC. Furthermore, decreased FA and increased RD were found in 15 WM regions, involving most of the WM tracts and predominance in the left hemisphere.

Considering that migraine features have been identified in the phenotype of persistent headache after COVID-19 resolution [3], we further compared COV patients with patients with episodic and chronic migraine, in order to investigate whether the pattern of the detected changes had elements in common with these two entities. According to our results, WM characteristics of persistent headache after COVID-19 are distinct than those in both types of migraine, while GM features are relatively similar to those of migraine, in a transitional situation between EM and HC.

In contrast to previous neuroimaging studies assessing subjects who recovered from COVID-19, this study includes patients with a specific persistent neurological manifestation: headache. Moreover, there is not only a comparison with healthy subjects, but also with patients with migraine, one of the most frequent better characterized primary headache disorders worldwide. Our eligibility criteria were very restrictive and selected a group of cases with no prior history of headache disorders, treatments with effect on the central nervous system or other neuropsychiatric or painful disorders. This set of comparisons allowed us to determine the extent and the specificity of the identified changes between COV patients and controls.

In comparison with other COVID-19 studies that were not focused on headache, our study showed FA changes in WM regions that have been identified in previous studies, particularly the corona radiata, the superior longitudinal fasciculus and the corpus callosum [19, 21, 22]. In our study, lower FA values were found in COV patients compared to HC. The same result was found in another study assessing patients who recovered from COVID-19 and suffered from anosmia during its acute phase [22]. Moreover, in this research and in our study, this result was identified in the superior longitudinal fasciculus. In one of the previous studies, Huang et al. reported lower FA values in patients admitted to an intensive care unit compared to patients who were not admitted [21]. In contrast, the study by Lu et al. showed the opposite FA trend, i.e., higher FA values in patients who recovered from COVID-19 compared to HC [19]. An important difference between this previous study and the present one is that the patients included in our research were still suffering from headache, while in the other study most patients were recovered, or some patients suffered olfactory loss as the exclusive persistent symptom. Our results may suggest that the severe situation in association with headache of the COV patients could cause medium- or long-term higher disorganization of the WM fiber bundles and their damage. Low FA values have been previously linked to demyelination, lower density, disorganization and changed membrane permeability [47–49]. Part of the results observed in our study could be related with COVID-19 infection and modulated or aggravated by headache persistence, given the similarity of the regions and the differences between our study and other prior studies. Specifically, these results may be related to anosmia as a concomitant syndrome that has been strongly associated with COVID-19 and which is frequent in patients with headache during the acute phase of COVID-19. The other parameter with differences between COV patients and HC was the RD, and higher values were found in the COV group. This result was also reported in the mentioned study assessing patients who recovered from COVID-19 accompanied by anosmia, also finding statistically significant differences in the superior longitudinal study [22]. Previously, Douaud et al. reported increased MD in patients who recovered from COVID-19 in diverse GM regions compared to controls, while in this study higher RD values in COV patients were identified in WM regions [20].

With regard to the extent of the identified WM changes, according to the effect size (Cohen’s *d* values), these changes were mild or moderate. Despite this modest magnitude, the trend of these changes is consistent with common findings in the neuroimage literature regarding changes detected in a wide range of pathologies, such as schizophrenia [50–52], Alzheimer’s disease [53–55], Parkinson’s disease [56–58], or migraine [11, 12, 18]. In these disorders, lower FA values and higher MD, RD or AD have been identified in the patient groups compared to healthy controls.

Regarding GM results, statistically significant differences were detected in a small group of regions in COV patients compared to HC. This result is in line with a recent study composed of hundreds of controls and patients who recovered from COVID-19, where the authors reported that GM changes between both groups were subtle [20]. Although thickness and GM volume differences have been reported in both studies, the identified regions with differences are distinct from one study to the other. This may imply that persistent headache affects a more specific group of regions in comparison to a generalized COVID-19 infection without a very specific long-term effect. Furthermore, in our study, no increased GM volume compared to controls was found in the COV patients, in contrast to a previous 3-months follow-up study [19]. In this follow-up study, the authors found increased volume in regions such as the olfactory cortex. As previously suggested, these differences between studies may be related to anosmia, especially considering that the inferior frontal lobe is associated with the olfactory cortex and the significant changes that were identified in regions like the pars orbitalis or the frontal lobe. However, a previous study assessing patients with COVID-19 and anosmia in its acute phase found no statistically significant GM volume changes, although the sample size was approximately the half in comparison to the present study [22].

To better determine the characteristics of headache in COV patients, we further compared GM and WM descriptors between these patients and patients with CM and EM. Regarding WM comparisons, taking the comparisons with controls as a reference, two main groups of results were detected. On the one hand, as in the comparison with HC, COV patients presented lower FA values than EM and CM patients. On the other hand, COV patients showed lower RD values than patients with CM and EM, except for a reduced group of regions with the opposite trend in the comparison with CM. In addition, COV patients presented lower AD and RD values than EM and CM. According to this pattern, COV patients would present some properties related to a situation similar but milder than EM. A hypothesis that may explain this situation is that changes in the aforementioned direction could be related to the effect of time, considering that patients with migraine have suffered from headaches a longer time compared to the COV patients. Moreover, it is interesting to note that FA and RD differences between COV patients and controls were found in the left hemisphere. On the other hand, lower MD and RD values in COV patients compared to migraine patients were identified in the right hemisphere, although higher RD values were also found mostly in few left hemisphere regions in COV patients compared to CM only after correcting the results for sex.

With regard to the additional differences found in the comparison between patient groups, and in line with previous studies with the same cohort of migraine patients, COV patients showed similar AD differences compared to those found in CM with respect to EM, with widespread lower AD values [18, 42, 46]. Thus, COV patients presented a situation similar to CM, with a possible intensified component of the axial diffusion. This effect suggests that the axial parameters could be highly related to the headache frequency. CM and COV patients present a high headache frequency, with 15 or more attacks per month and almost every day, respectively [9]. In fact, in the sample employed in this study, all COV patients suffered from daily headache. On the contrary, patients with EM, especially excluding patients with high-frequency EM (more than eight headache days per month), presented opposite values in comparison with the other two headache groups. This effect is in line with the latest migraine studies assessing AD in patients with EM [16, 18, 59, 60]. Thus, in relation to the almost daily headache attacks of COV patients, the permanent activation of the brain can cause specific changes possibly associated with axonal damage or loss, or short-term demyelination [48, 61–63]. Considering the differences between the headache groups, we expected differences in AD between COV patients and HC, but we obtained no statistically significant results. The reason of this lack of differences may be associated with the higher age of controls in this study, which may be a key factor in the changes of diffusion in the main or axonal direction. Nevertheless, to consider the potential differences caused by the age difference, results were corrected by age. In healthy adults, it has been reported that AD significantly changes with age. In regions such as the corona radiata, which presented statistically significant differences in COV patients according to our results, AD has been reported to decrease with age in adults [64]. This result seems to be in line with our correlation results in COV patients, as we found a negative correlation between disease duration and mean AD values in the posterior corona radiata. However, the opposite results have also been reported in GM and other WM regions such as the fornix [64, 65].

GM results showed a similar set of differences in comparison with the described RD and MD differences in WM, i.e., a situation analogous but milder than EM. In contrast to a previous study that identified differences morphometry parameters between patients with migraine (EM and CM) and controls in diverse regions, statistically significant differences were detected in a small group of regions [12]. The number of regions with significant differences was particularly small between EM and COV patients. Since COV patients present a smaller degree of changes with respect to HC, the effect of time may be important in GM changes.

Notable limitations are present in this study. First, there were no baseline MRI acquisitions of the COV patients. This implies that we were unable to observe the effects of the persistent headache before, during and after the infection to determine the changes associated with each pathophysiological mechanism. To compensate the lack of longitudinal data, we compared the main group of interest not only with healthy controls, but also with the two currently distinguished migraine types, EM and CM. However, patients with migraine, especially EM, are younger than COV patients. Despite all comparisons with migraine groups were corrected for age, this factor could have influenced the results. Moreover, due to the age difference between the patient groups, the controls included in this study were older with respect to previous studies where the target was the comparison with patients with migraine [12, 18, 42]. The remarkable difference of age of the control groups might have altered the established hypothesis regarding the differences of the diverse parameters between controls and each patient group. We preferred to follow the results from previous studies with the same patients to avoid any potential bias in the results caused for employing controls not balanced for age and sex in comparison with patients with migraine. Considering the diverse phenotypes of headache in the patients who recovered from COVID-19, an assessment of the differences between the migraine and control groups and each long-term headache type was not conducted. The reason was that we preferred to preserve a group with a relatively high sample size, as changes in headache disorders have been reported to be subtle and therefore a small sample size can be insufficient to identify statistically significant differences. Due to the lack of inflammation biomarkers at the diverse timepoints of the follow-up period, we could not clearly establish whether changes are strongly associated with the COVID-19 infection or more related to headache. In addition, no T2-weighted or FLAIR images were available in either the controls or the patients with migraine. Considering the potential relationship between WM hyperintensities and WM integrity, and also the identification of these findings in patients with migraine [66–68], we were unable to assess a possible factor that might influence the results. Finally, since recruitment of the COV group was performed early in the COVID-19 pandemic evolution, no generalization can be made with regard to the possible effects of other COVID-19 variants such as delta or omicron, or the effect of vaccination or reinfection.

## Conclusions

Patients with persistent headache after COVID-19 resolution showed diverse changes in GM and WM structure. Changes in GM were subtle and affected anterior areas, including the pars orbitalis, the fusiform gyrus and the frontal pole. On the one hand, the observed WM changes were diffuse, involved most of the WM tracts and seemed related to the impairment of WM fiber bundles. On the other hand, the WM changes presented a situation analogous but milder than migraine. Future studies are needed to discriminate long-term and evolving changes associated with COVID-19 infection and headache to assess the differences between long-term headache phenotypes after COVID-19 recovery.

## Supplementary Information

Below is the link to the electronic supplementary material.

Supplementary file1 (PDF 301 KB)

## Data Availability

The data that supports the findings of this study are available from the corresponding author, upon reasonable request.

## Abbreviations

AD: Axial diffusivity
ANCOVA: Analysis of covariance
CM: Chronic migraine
COVID-19: Coronavirus disease 2019
COV: Group of patients with persistent headache after COVID-19 resolution
DTI: Diffusion tensor imaging
DWI: Diffusion-weighted imaging
EM: Episodic migraine
FA: Fractional anisotropy
FDR: False discovery rate
FEW: Family wise error
GM: Gray matter
HC: Healthy controls
ICHD-3: International Classification of Headache Disorders, 3rd edition
MD: Mean diffusivity
MNI: Montreal Neurological Institute
RD: Radial diffusivity
RT-PCR: Reverse-transcriptase-polymerase-chain-reaction
TBSS: Tract-based spatial statistics
TE: Echo time
TFCE: Threshold-free cluster enhancement
TI: Inversion time
TR: Repetition time
WM: White matter
