# Supplementary material for: Structural brain changes in patients with persistent headache after COVID-19 resolution
Source: J Neurol. 2022 Sep 30;270(1):13–31. doi: 10.1007/s00415-022-11398-z (PMC9522538; doi:10.1007/s00415-022-11398-z)
Supplement: Supplementary file 1 — Supplementary file1 (PDF 301 KB) [file 415_2022_11398_MOESM1_ESM.pdf]

Table S1. Gray matter regions with gray matter morphometry statistically significant differences between patients with persistent headache after COVID-19 resolution and healthy controls (HC).

Gray matter region and parameter	COVID-19 headache (n=42)	HC (n=43)	Statistical test
**Cortical thickness (mm)**			
Right pars orbitalis	2.55 ± 0.15	2.67 ± 0.13	t = -3.96, p = 0.008†
Left rostral anterior cingulate gyrus*	2.73 ± 0.17	2.85 ± 0.17	F(1,81) = 12.37, p = 0.024
**Gray matter volume (mm^3)**			
Left pars orbitalis	2161 ± 338	2395 ± 366	F(1,82) = 11.97, p = 0.029
Right pars orbitalis	2523 ± 331	2767 ± 455	F(1,82) = 10.22, p = 0.033
Right fusiform gyrus	8346 ± 1023	9002 ± 1081	F(1,82) = 9.39, p = 0.033
Right frontal pole	1001 ± 157	1103 ± 161	F(1,82) = 9.94, p = 0.033

†Two-tailed, unpaired Student's t-test for equal variances. Data are expressed as mean ± SD. All gray matter volume comparisons were carried out using an ANCOVA, adjusting the results by the total intracranial volume. All p-values are adjusted following a False Discovery Rate procedure. *The left rostral anterior cingulate gyrus showed statistically significant differences when adding age and sex as covariates.

Table S2. Gray matter regions with subcortical gray matter volume statistically significant differences between patients with persistent headache after COVID-19 resolution and patients with episodic migraine (EM).

Gray matter region and parameter	COVID-19 headache (n=42)	EM (n=43)	Statistical test
**Cortical thickness (mm)**			
Left paracentral lobule	2.46 ± 0.15	2.34 ± 0.14	$F(1,82) = 14.68$, $p = 0.008$
**Gray matter volume (mm^3)**			
Left accumbens	475 ± 62	494 ± 92	$F(1,81) = 9.57$, $p = 0.022$
Right thalamus	6593 ± 624	6882 ± 788	$F(1,81) = 9.27$, $p = 0.025$

Data are expressed as mean ± SD. All gray matter volume comparisons were carried out using an ANCOVA, adjusting the results by the total intracranial volume and age. All p-values are adjusted following a False Discovery Rate procedure.

Table S3. Gray matter regions with gray matter morphometry statistically significant differences between patients with persistent headache after COVID-19 resolution and patients with chronic migraine (CM).

Gray matter region and parameter	COVID-19 headache (n=42)	CM (n=43)	Statistical test
**Cortical curvature (mm^{-1})**			
Left cuneus	0.142 ± 0.008	0.149 ± 0.009	F(1,82) = 15.01, p = 0.007
Right precuneus	0.125 ± 0.006	0.130 ± 0.006	F(1,82) = 12.04, p = 0.028
**Cortical thickness (mm)**			
Left banks of the superior temporal sulcus	2.51 ± 0.14	2.40 ± 0.13	F(1,82) = 13.51, p = 0.007
Left paracentral lobule	2.46 ± 0.15	2.34 ± 0.16	F(1,82) = 14.17, p = 0.007
**Gray matter volume (mm^3)**			
Left caudal middle frontal gyrus	6104 ± 950	5455 ± 850	F(1,81) = 13.89, p = 0.012
Left paracentral lobule	3430 ± 382	3159 ± 434	F(1,81) = 10.30, p = 0.032
Left posterior cingulate gyrus	3120 ± 597	2825 ± 391	F(1,81) = 9.14, p = 0.038

Data are expressed as mean ± SD. All comparisons were carried out using an ANCOVA, adjusting the results by the total intracranial volume. All p-values are adjusted following a False Discovery Rate procedure.

Table S4. White matter regions from the ICBM-DTI-81 White Matter Atlas for which significant decreased FA values were found in patients with persistent headache after COVID-19 compared to HC.

White Matter tract	Minimum p-value (FWE-corrected)	Volume (mm^3)
Superior longitudinal fasciculus L	.031	47
Body of corpus callosum	.034	103
Splenium of corpus callosum	.034	423
Anterior corona radiata R/L	.042/.030	230/196
Superior corona radiata L	.029	471
Posterior corona radiata L	.028	145
External capsule L	.028	392
Posterior limb of internal capsule L	.022	529
Retrolenticular part of internal capsule L	.025	513
Anterior limb of internal capsule L	.042	120
Sagittal stratum L	.030	211
Posterior thalamic radiation L	.027	132
Cerebral peduncle L	.026	170
Corticospinal tract L	.046	122

FWE = Family-wise error; L = left.

The column Volume represents the volume from the atlas region with significant results. No regions with volume equal or lower than 30 mm^3 were included in this Table. All these regions presented statistically significant differences when adding sex as covariate.

Table S5. Additional white matter regions from the ICBM-DTI-81 White Matter Atlas for which significant decreased FA values were found in patients with persistent headache after COVID-19 compared to HC when adding sex as a covariate.

White Matter tract	Minimum p-value (FWE-corrected)	Volume (mm^3)
Genu of corpus callosum	.029	208
Superior corona radiata R	.040	257
Posterior corona radiata R	.042	109
External capsule R	.038	303
Anterior limb of internal capsule R	.038	200
Posterior limb of internal capsule R	.044	52
Superior fronto-occipital fasciculus R	.044	31

FWE = Family-wise error; L = left.

The column Volume represents the volume from the atlas region with significant results. No regions with volume equal or lower than 30 mm^3 were included in this Table.

Table S6. White matter regions from the ICBM-DTI-81 White Matter Atlas for which significant increased RD values were found in patients with persistent headache after COVID-19 compared to HC.

White Matter tract	Minimum p-value (FWE-corrected)	Volume (mm^3)
Superior longitudinal fasciculus L	.042	155
Superior corona radiata L	.043	367
Posterior limb of internal capsule L	.045	123

FWE = Family-wise error; L = left; R = right.

The column Volume represents the volume from the atlas region with significant results. No regions with volume equal or lower than 30 mm^3 were included in this Table. All these regions presented statistically significant differences when adding sex as covariate.

Table S7. Additional white matter regions from the ICBM-DTI-81 White Matter Atlas for which significant increased RD values were found in patients with persistent headache after COVID-19 compared to HC when adding sex as a covariate.

White Matter tract	Minimum p-value (FWE-corrected)	Volume (mm^3)
Anterior corona radiata R/L	.047/.036	102/214
Posterior corona radiata L	.035	67
Anterior limb of internal capsule L	.040	96
Retrolenticular part of internal capsule L	.040	336
Sagittal stratum L	.043	102
Cerebral peduncle L	.042	49

FWE = Family-wise error; L = left; R = right.

The column Volume represents the volume from the atlas region with significant results. No regions with volume equal or lower than 30 mm^3 were included in this Table.

Table S8. White matter regions from the ICBM-DTI-81 White Matter Atlas for which significant decreased AD values were found in patients with persistent headache after COVID-19 compared to EM.

White Matter tract	Minimum p-value (FWE-corrected)	Volume (mm^3)
Middle cerebellar peduncle	< .001	2502
Inferior cerebellar peduncle R/L	< .001/< .001	188/140
Superior cerebellar peduncle R/L	< .001/< .001	168/156
Superior longitudinal fasciculus R/L	< .001/.002	1311/934
Genu of corpus callosum	.001	827
Body of corpus callosum	< .001	1069
Splenium of corpus callosum	< .001	154
Anterior corona radiata R/L	< .001/< .001	563/724
Superior corona radiata R	< .001	1041
Posterior corona radiata R/L	< .001/.002	277/189
External capsule R/L	< .001/< .001	975/524
Posterior limb of internal capsule R/L	< .001/.002	833/561
Retrolenticular part of internal capsule R/L	< .001/< .001	696/680
Anterior limb of internal capsule R/L	< .001/< .001	478/395
Sagittal stratum R/L	< .001/< .001	622/577
Posterior thalamic radiation R/L	< .001/< .001	729/765
Cerebral peduncle R/L	< .001/< .001	402/311
Corticospinal tract R/L	< .001/< .001	266/164
Pontine crossing tract	< .001	323
Fornix (cres) R/L	< .001/.003	310/301
Medial lemniscus R/L	< .001/< .001	68/66
Tapetum R	< .001	67

Cingulum (hippocampus) R	.002	178
Superior fronto-occipital fasciculus R	< .001	46

FWE = Family-wise error; L = left; R = right.

The column Volume represents the volume from the atlas region with significant results. No regions with volume equal or lower than 30 mm^3 were included in this Table. All these regions presented statistically significant differences when adding sex as covariate.

Table S9. White matter regions from the ICBM-DTI-81 White Matter Atlas for which significant decreased FA values were found in patients with persistent headache after COVID-19 compared to EM.

White Matter tract	Minimum p-value (FWE-corrected)	Volume (mm^3)
Superior longitudinal fasciculus L	.044	156
Genu of corpus callosum	.038	199
Body of corpus callosum	.038	841
Anterior corona radiata R/L	.038/.042	36/123
Superior corona radiata L	.042	310
Posterior corona radiata L	.045	86
External capsule L	.022	207
Posterior limb of internal capsule L	.020	171
Retrolenticular part of internal capsule L	.019	597
Sagittal stratum L	.022	391
Posterior thalamic radiation L	.019	134
Cerebral peduncle L	.028	181
Fornix (cres) L	.019	291
Fornix (column and body)	.044	134

FWE = Family-wise error; L = left; R = right.

The column Volume represents the volume from the atlas region with significant results. No regions with volume equal or lower than 30 mm^3 were included in this Table. All these regions presented statistically significant differences when adding sex as covariate.

Table S10. Additional white matter regions from the ICBM-DTI-81 White Matter Atlas for which significant decreased FA values were found in patients with persistent headache after COVID-19 compared to EM when adding sex as a covariate.

White Matter tract	Minimum p-value (FWE-corrected)	Volume (mm^3)
Splenium of corpus callosum	.035	149
Superior corona radiata R	.032	204
Posterior corona radiata R	.043	51
Uncinate fasciculus L	.018	34
Cingulum (hippocampus) R	.047	97

FWE = Family-wise error; L = left; R = right.

The column Volume represents the volume from the atlas region with significant results. No regions with volume equal or lower than 30 mm^3 were included in this Table.

Table S11. White matter regions from the ICBM-DTI-81 White Matter Atlas for which significant decreased MD values were found in patients with persistent headache after COVID-19 compared to EM.

White Matter tract	Minimum p-value (FWE-corrected)	Volume (mm^3)
**Middle cerebellar peduncle**	.004	2201
**Inferior cerebellar peduncle R**/L	.004/.028	174/107
**Superior cerebellar peduncle R**/L	.005/.005	110/45
**Superior longitudinal fasciculus R**	< .001	1122
Genu of corpus callosum	.029	265
**Splenium of corpus callosum**	.005	342
**Anterior corona radiata R**	.015	429
**Superior corona radiata R**	< .001	715
**Posterior corona radiata R**	.001	238
**External capsule R**	.001	1033
**Posterior limb of internal capsule R**	< .001	906
**Retrolenticular part of internal capsule R**	< .001	699
**Anterior limb of internal capsule R**	< .001	451
**Sagittal stratum R**	< .001	611
**Posterior thalamic radiation R**	< .001	597
**Cerebral peduncle R**	< .001	362
**Corticospinal tract R/L**	.005/.006	254/82
**Pontine crossing tract**	.005	282
**Fornix (cres) R**	< .001	274
**Medial lemniscus R/L**	.004/.005	86/58
**Tapetum R**	.003	37

FWE = Family-wise error; L = left; R = right.

The column Volume represents the volume from the atlas region with significant results. No regions with volume equal or lower than 30 mm^3 were included in this Table. Regions in bold also showed statistically significant differences when adding sex as covariate.

Table S12. White matter regions from the ICBM-DTI-81 White Matter Atlas for which significant decreased RD values were found in patients with persistent headache after COVID-19 compared to EM.

White Matter tract	Minimum p-value (FWE-corrected)	Volume (mm^3)
Middle cerebellar peduncle	.023	1093
Inferior cerebellar peduncle R	.025	86
Superior cerebellar peduncle R	.022	50
Superior longitudinal fasciculus R	.012	76
Splenium of corpus callosum	.048	62
Superior corona radiata R	.012	289
Posterior corona radiata R	.017	89
External capsule R	.014	749
Posterior limb of internal capsule R	.012	825
Retrolenticular part of internal capsule R	.012	538
Anterior limb of internal capsule R	.012	193
Sagittal stratum R	.013	431
Posterior thalamic radiation R	.013	279
Cerebral peduncle R	.015	235
Corticospinal tract R	.022	164
Pontine crossing tract	.021	112
Fornix (cres) R	.021	112
Uncinate fasciculus R	.043	34

FWE = Family-wise error; L = left; R = right.

The column Volume represents the volume from the atlas region with significant results. No regions with volume equal or lower than 30 mm^3 were included in this Table.

Table S13. White matter regions from the ICBM-DTI-81 White Matter Atlas for which significant decreased AD values were found in patients with persistent headache after COVID-19 compared to CM.

White Matter tract	Minimum p-value (FWE-corrected)	Volume (mm^3)
Middle cerebellar peduncle	.031	310
Inferior cerebellar peduncle R	.039	41
Superior longitudinal fasciculus R	.012	203
External capsule R	.007	581
Posterior limb of internal capsule R	.008	587
Retrolenticular part of internal capsule R/L	.008/.033	393/37
Anterior limb of internal capsule R	.011	227
Sagittal stratum R/L	.008/.033	340/111
Posterior thalamic radiation R/L	.008/.028	118/224
Cerebral peduncle R	.008	258
Fornix (cres) R	.007	251

FWE = Family-wise error; L = left; R = right.

The column Volume represents the volume from the atlas region with significant results. No regions with volume equal or lower than 30 mm^3 were included in this Table. All these regions presented statistically significant differences when adding sex as covariate.

Table S14. White matter regions from the ICBM-DTI-81 White Matter Atlas for which significant decreased FA values were found in patients with persistent headache after COVID-19 compared to CM.

White Matter tract	Minimum p-value (FWE-corrected)	Volume (mm^3)
Superior longitudinal fasciculus L	.036	39
Genu of corpus callosum	.020	467
Body of corpus callosum	.020	1043
Splenium of corpus callosum	.026	664
Anterior corona radiata R/L	.041/.021	55/295
Superior corona radiata L	.024	498
Posterior corona radiata L	.024	161
External capsule L	.013	660
Posterior limb of internal capsule L	.013	295
Retrolenticular part of internal capsule L	.010	570
Anterior limb of internal capsule L	.040	135
Sagittal stratum L	.012	310
Posterior thalamic radiation L	.014	102
Cerebral peduncle L	.032	159
Fornix (cres) L	.009	366
Fornix (column and body)	.025	142

FWE = Family-wise error; L = left; R = right.

The column Volume represents the volume from the atlas region with significant results. No regions with volume equal or lower than 30 mm^3 were included in this Table.

Table S15. Additional white matter regions from the ICBM-DTI-81 White Matter Atlas for which significant decreased FA values were found in patients with persistent headache after COVID-19 compared to CM when adding sex as a covariate.

White Matter tract	Minimum p-value (FWE-corrected)	Volume (mm^3)
Tapetum L	.025	33
Superior fronto-occipital fasciculus L	.025	64

FWE = Family-wise error; L = left; R = right.

The column Volume represents the volume from the atlas region with significant results. No regions with volume equal or lower than 30 mm^3 were included in this Table.

Table S16. White matter regions from the ICBM-DTI-81 White Matter Atlas for which significant decreased MD values were found in patients with persistent headache after COVID-19 compared to CM.

White Matter tract	Minimum p-value (FWE-corrected)	Volume (mm^3)
Middle cerebellar peduncle	.026	928
Inferior cerebellar peduncle R	.026	103
**Superior longitudinal fasciculus R**	.011	191
**Superior corona radiata R**	.009	208
**External capsule R**	.011	365
**Posterior limb of internal capsule R**	.008	758
**Retrolenticular part of internal capsule R**	.008	580
**Sagittal stratum R**	.009	450
**Posterior thalamic radiation R**	.009	158
**Cerebral peduncle R**	.008	193
Corticospinal tract R	.040	146
Pontine crossing tract	.041	81
**Fornix (cres) R**	.008	203

FWE = Family-wise error; L = left; R = right.

The column Volume represents the volume from the atlas region with significant results. No regions with volume equal or lower than 30 mm^3 were included in this Table. Regions in bold also showed statistically significant differences when adding sex as covariate.

Table S17. White matter regions from the ICBM-DTI-81 White Matter Atlas for which significant increased RD values were found in patients with persistent headache after COVID-19 compared to CM when adding sex as a covariate.

White Matter tract	Minimum p-value (FWE-corrected)	Volume (mm^3)
Superior longitudinal fasciculus L	.025	408
Body of corpus callosum	.026	87
Anterior corona radiata L	.038	159
Superior corona radiata L	.025	663
External capsule L	.029	58
Posterior limb of internal capsule L	.028	169
Retrolenticular part of internal capsule L	.037	74

FWE = Family-wise error; L = left; R = right.

The column Volume represents the volume from the atlas region with significant results. No regions with volume equal or lower than 30 mm^3 were included in this Table.

Table S18. White matter regions from the ICBM-DTI-81 White Matter Atlas for which significant decreased RD values were found in patients with persistent headache after COVID-19 compared to CM.

White Matter tract	Minimum p-value (FWE-corrected)	Volume (mm^3)
Superior corona radiata R	.034	216
Posterior corona radiata R	.035	53
External capsule R	.036	146
Posterior limb of internal capsule R	.031	490
Retrolenticular part of internal capsule R	.032	404
Sagittal stratum R	.041	306
Posterior thalamic radiation R	.037	126
Cerebral peduncle R	.035	65
Fornix (cres) R	.034	72

FWE = Family-wise error; L = left; R = right.

The column Volume represents the volume from the atlas region with significant results. No regions with volume equal or lower than 30 mm^3 were included in this Table.

Table S19. White matter regions from the ICBM-DTI-81 White Matter Atlas for which significant increased RD values were found in patients with persistent headache after COVID-19 compared to CM.

White Matter tract	Minimum p-value (FWE-corrected)	Volume (mm^3)
Superior longitudinal fasciculus L	.042	204
Genu of corpus callosum	.042	89
Body of corpus callosum	.042	466
Splenium of corpus callosum	.048	141
Superior corona radiata L	.040	334
Posterior corona radiata L	.043	67

FWE = Family-wise error; L = left; R = right.

The column Volume represents the volume from the atlas region with significant results. No regions with volume equal or lower than 30 mm^3 were included in this Table. All these regions presented statistically significant differences when adding sex as covariate.

Table S20. Additional white matter regions from the ICBM-DTI-81 White Matter Atlas for which significant increased RD values were found in patients with persistent headache after COVID-19 compared to CM when adding sex as a covariate.

White Matter tract	Minimum p-value (FWE-corrected)	Volume (mm^3)
Anterior corona radiata L	.028	231
External capsule L	.033	278
Anterior limb of internal capsule L	.028	79
Retrolenticular part of internal capsule L	.049	71
Fornix (cres) L	.048	56
Superior fronto-occipital fasciculus L	.028	51

FWE = Family-wise error; L = left; R = right.

The column Volume represents the volume from the atlas region with significant results. No regions with volume equal or lower than 30 mm^3 were included in this Table.
